# Risk factors for readmission after ureteroscopy for stone disease: Modern single centre experience

**DOI:** 10.1002/bco2.70007

**Published:** 2025-03-23

**Authors:** Shuhei Hirano, Margaret A. Knoedler, Shuang Li, Emily C. Serrell, Ali S. Antar, Stephen Y. Nakada

**Affiliations:** ^1^ Department of Urology University of Wisconsin School of Medicine and Public Health Madison WI USA

**Keywords:** ED visits, laser lithotripsy, readmission, ureteroscopy, urinary stone disease

## Abstract

**Objectives:**

To identify factors that increase a patient's risk of readmission in the immediate postoperative period following ureteroscopy.

**Materials and Methods:**

An IRB‐approved surgical database of patients with renal and ureteral stones at a single institution was retrospectively analysed for patients who underwent ureteroscopies and had 30 days follow‐up from September 2016 to June 2019. We reviewed the most recent 600 cases (300 consecutive women and 300 consecutive men). Patient characteristics including gender, body mass index (BMI) and comorbidities (hypertension, gout, diabetes mellitus (DM), recurrent urinary tract infections (UTIs), chronic kidney disease (CKD), bowel disease), history of preoperative ED visit and surgical factors (preoperative stent, stone size) were used to conduct univariate and multivariable logistic regression analysis. Outcome measures included readmission within 30 days postoperatively. Exclusion criteria included age <18 and <30 days follow‐up.

**Results:**

Of the 600 patients, 40 (6.7%) visited the ED and 16 (2.7%) were admitted within 30 days postoperatively. None of the patient characteristics or surgical factors we examined were associated with ED visits postoperatively (all p > 0.05). Patients were more likely to have a postoperative admission if they were older (age 68 ± 15 vs 56 ± 15, p < 0.002; OR 1.06; 95% CI 1.01–1.10, p = 0.01) or had a history of recurrent UTIs (OR 7.40, 95%CI 1.78–30.67, p = 0.006). No other factors correlated with postoperative admissions.

**Conclusions:**

Older age and history of recurrent UTIs increased patients' risks of readmission within 30 days of ureteroscopy. This finding is particularly important when hospital beds are at a premium. Older patients and patients with recurrent UTIs should be targeted for preoperative interventions to prevent readmission.

## 1 INTRODUCTION

Urinary stone disease is a common disease globally with rates ranging from 5 to 9% in Europe, and 1–5% in Asia.^1^ According to data from the National Health and Nutrition Examination Survey, patients' self‐reported history of stone disease was estimated to be approximately 10% in the United States.^2^

This has led to an expansion of indications for ureteroscopy (URS) and laser lithotripsy and the development of technological surgical advances and digital equipment.^3^ The European Association of Urology (EAU) guidelines recommend URS as a safe, first‐line procedure in most urinary stone disease patients. The American Urological Association recommends URS as first‐line therapy for mid and distal ureteral stones.^4^, ^5^ Although URS is an established minimally invasive procedure for the diagnosis and treatment of urinary stone disease, there has been a rise in reported deaths over the past decade.^6^ While many studies have reported complications and mortality of urinary stone lithotripsy,^6^, ^7^, ^8^ few have identified the need for readmission after URS.^9^, ^10^ Understanding the risk factors associated with readmission and emergency department (ED) visits may lead to decreased morbidity in these patients.

Moreover, hospital readmissions are increasingly scrutinized as a preventable source of health care spending. Unplanned readmission exacts an economic toll, with unplanned episodes of care after stone surgery exceeding an average expenditure of $20 000–30 000 per patient in the US.^11^ Under the Hospital Readmissions Reduction Program, the Centers for Medicare and Medicaid Services have begun adjusting payments to hospitals based on readmission rates since 2012. Stone et al noted that this practice is likely to grow to include urologic surgeries.^12^ Although some studies have described unplanned ED visits or readmission rates following stone procedures, the clinical factors contributing to unplanned ED visits and readmission after URS remain unclear.^13^, ^14^ Especially, when inpatient beds and hospital resources are scarce, minimizing ED visits and potential hospital admission is desirable.

Postoperative infections represent one of the biggest reasons for readmission after URS. Veeratterapillay et al reported that 6.8% of patients undergoing URS had a hospital diagnosis of infection, and urinary tract infections (UTIs), stent symptoms and abdominal pain were the most common indications for admission within 30 days in the UK.^15^ Bloom et al showed that the patients who had two or more comorbidities and complications, including urosepsis, were more likely to be readmitted after URS.^9^ However, there is a lack of data on how infection relates to the rate of visiting ED and readmission after URS.

Our objective was to identify factors that increase a patient's risk of ED visits and readmission in the immediate postoperative period following URS.

## 2 MATERIALS AND METHODS

### 2.1 Patients

From an IRB‐approved surgical database, we collected data from the most recent patients with renal and ureteral stones at a single centre and retrospectively identified those who underwent URS with laser lithotripsy and had 30 days follow‐up. We reviewed the most recent 600 patients as an equal number of cases from each gender (300 consecutive females from March 2016 to June 2016 and 300 consecutive males from June 2016 to June 2019) separately to assess their differences regarding readmission following the procedure. No statistical sample size calculations were conducted because we predicted that the event size was small in this retrospective study. All emergency surgeries and patients admitted to the hospital directly following surgery were excluded. For patients with staged procedures, the first procedure was considered the index stone procedure. Urine cultures were routinely tested preoperatively and patients with bacteriuria were prescribed a short course of culture‐appropriate pre‐operative antibiotics. All patients receive prophylactic antibiotics in the operating room.

A standardized URS procedure was performed for all patients by 13 experienced urologists. A semirigid ureteroscope was used for distal ureteral stones and a flexible ureteroscope was used for stones in the proximal ureter, renal pelvis or calyces. A safety wire was used routinely for ureteral stones, but not during treatment of stones in the kidney. A ureteral access sheath (UAS) is not routinely used during URS at our institution. Ureteral stents are most often placed after URS, and we removed them in the clinic cystoscopically 5–7 days postoperatively.

Stone size was determined by preoperative imaging. Patient characteristics including age, gender, race, body mass index (BMI), comorbidities (hypertension, gout, diabetes mellitus (DM), recurrent UTIs, chronic kidney disease (CKD), bowel disease), history of preoperative ED visit, surgical factors (preoperative stent, stone size, surgery time) and complications were evaluated. Surgery time was defined as the time between insertion and removal of the instrument. We defined recurrent UTIs as “recurrences of uncomplicated and/or complicated UTIs, with a frequency of at least three UTIs/year or two UTIs in the last 6 months” according to EAU guideline.^16^ Outcome measures included ED visits and readmission within 30 days postoperatively. Exclusion criteria included age <18 and <30 days follow‐up. Seven patients were excluded due to lost follow‐up within 30 days after URS. The study protocol was conducted in accordance with the guidelines of the Declaration of Helsinki and was approved by the Institutional Review Board of the University of Wisconsin School of Medicine and Public Health (no. 2014–0033).

### 2.2 Statistical analysis

We compared patients who had an ED visit to those who did not have an ED visit within 30 days of surgery and also looked at patients admitted to the hospital within 30 days of surgery using chi‐square (categorical variables) and T‐test (continuous variables). We used both univariate and multivariable analyses while controlling for demographic and clinical factors. Multivariable analysis was performed using logistic regression to compare the factor of readmission. Based on previous studies, in addition to the result of univariable analysis, we selected predictors for the modelling, including gender, BMI, preoperative ED visits, stone size and several comorbid conditions (diabetes, recurrent UTIs and CKD) hypothesized to potentially affect outcomes^8^, ^10^ Statistical significance was set at p < 0.05. All reported p‐values were two‐sided. SPSS Version 25.0 (IBM Corp., Armonk, NY, USA) was used for all data analysis.

## 3 RESULTS

Patients were 56 ± 15 years old with a mean BMI of 31 ± 7.9 kg/m^2^. Age, BMI and race did not differ between sexes (Table 1). The proportion of female to male patients was not different for other races. The pre‐operative stone size was 8.5 ± 4.7 mm. The most common indications for preoperative stenting were pain and infection. Female patients were more likely to have a preoperative stent than male patients (24% vs 14%; P = 0.002). Furthermore, there were significantly more females with recurrent UTIs compared to males (7.7% vs 2.7%; P = 0.006), and males were more likely to have gout than females (7.3% vs 2.3%; P = 0.004, respectively).

**TABLE 1:** Characteristic of the study population (sex difference).

	Overall (n = 600)	Male (n = 300)	Female (n = 300)	P
Age (years)	56 ± 15	57 ± 15	55 ± 15	0.172
BMI (kg/m^2^)	31 ± 7.9	31 ± 7.7	31 ± 8.1	0.543
Race (4 declined)				0.119
White	562 (94%)	282 (95%)	280 (94%)	
African American	15 (2.5%)	4 (1.3%)	11 (3.7%)	
Asian	13 (2.2%)	9 (3.0%)	4 (1.3%)	
Native American	6 (1.0%)	2 (0.7%)	4 (1.3%)	
Preoperative stone size (mm)	8.5 ± 4.7	8.3 ± 4.1	8.8 ± 5.3	0.255
Pre‐stented				0.002
Pre‐stented	115 (19%)	43 (14%)	72 (24%)	
Nephrostomy tube placed	11 (1.8%)	3 (1.0%)	8 (2.7%)	
Surgery time (minutes)	44.3 ± 34.4	45.9 ± 31.3	42.8 ± 37.2	0.274
Visited ED within 30 days	40 (6.7%)	20 (6.7%)	20 (6.7%)	1
Visited ED and admitted	16 (2.7%)	9 (3.0%)	7 (2.3%)	0.612
Preoperative ED visit	224 (37%)	114 (38%)	110 (37%)	0.736
Comorbidities				
Diabetes mellitus	134 (22%)	74 (25%)	60 (20%)	0.17
Hypertension	256 (43%)	139 (46%)	117 (39%)	0.069
Gout	29 (4.8%)	22 (7.3%)	7 (2.3%)	0.004
Recurrent UTIs	31 (5.2%)	8 (2.7%)	23 (7.7%)	0.006
Bowel disease	133 (22%)	58 (19%)	75 (25%)	0.095
Chronic kidney disease	52 (8.7%)	24 (8.0%)	28 (9.3%)	0.562
Indication of readmissions:				0.567
UTIs	7 (44%)	5 (56%)	2 (29%)	
Flank pain	3 (19%)	2 (22%)	1 (14%)	
Unclassified fever	2 (13%)	1 (11%)	1 (14%)	
Others: stent displacement, acute kidney disease, chest pain and chronic heart failure	4 (25%)	1(11%)	3 (43%)	

Of the 600 patients, 40 (6.7%) visited ED and 16 (2.7%) were admitted within 30 days postoperatively. The indications for ED visits were pain (n = 25, 63%), UTI (n = 5, 13%) and other (fever, haematuria, stent displacement) (n = 10, 25%). Reasons for admission of the remaining 16 patients were UTIs including urosepsis (n = 7, 44%), flank pain (n = 3, 19%), unclassified fever (n = 2,13%), acute kidney disease (n = 1, 6.2%), stent displacement (n = 1, 6.2%) and other symptoms (chest pain and chronic heart failure) (n = 2, 13%). When looking at the difference between patients without ED visits and those with ED visits, none of the patient characteristics or surgical factors examined were associated with ED visits postoperatively (all p > 0.05 Table S1).

When we compared admitted patients with non‐admitted patients, the age of admitted patients was higher than that of non‐admitted patients (68 ± 15 vs 56 ± 15, p = 0.002, Table 2). In addition, there were more patients with recurrent UTIs and chronic kidney diseases in the admitted patient group than in not admitted patient group (25% vs 4.6%, p < 0.001; and 25% vs 8.2%, p = 0.019, respectively). A total of 134 patients (22%) had a diagnosis of DM, but this was not significantly different between the admitted vs. non‐admitted patients. BMI, race, ethnicity, preoperative stone size and preoperative stenting also did not differ between the two groups. Moreover, admitted patients had significantly higher rates of hypertension and a history of recurrence UTIs compared to not admitted patients who visited ED (63% vs 21% p = 0.008, 25% vs 0% p = 0.01, respectively, Table S2). The mean age in admitted patients is significantly higher than in not admitted patients who visited ED, similar to Table 2 (68 ± 15 vs 48 ± 17, p < 0.001). Not admitted patients who visited ED had significantly higher rates of preoperative ED visits (63% vs 19%, p = 0.006).

**TABLE 2:** Comparison of the patients who did not need admission with patients who needed admission.

	Overall (n = 600)	Not admitted (n = 584)	Admitted (n = 16)	P
Age (years)	56 ± 15	56 ± 15	68 ± 15	0.002
BMI (kg/m^2^)	31 ± 7.9	31 ± 7.9	28 ± 6.9	0.112
Race				0.697
White	562 (94%)	547 (94%)	15 (94%)	
African American	15 (2.5%)	14 (2.4%)	1 (6.3%)	
Asian	13 (2.2%)	13 (2.2%)	0 (0%)	
Native American	6 (1.0%)	6 (1.0%)	0 (0%)	
Preoperative stone size (mm)	8.5 ± 4.7	8.6 ± 4.7	7.8 ± 3.1	0.532
Preoperative ED visit	224 (37%)	221 (38%)	3 (19%)	0.119
Pre stented				0.168
Pre‐stented	115 (19%)	110 (19%)	5 (31%)	
Nephrostomy tube placed	11 (1.8%)	10 (1.7%)	1 (6.3%)	
Surgery time (minutes)	44 ± 34	44 ± 35	42 ± 16	0.758
Comorbidities				
Diabetes mellitus	134 (22%)	131 (22%)	3 (19%)	0.727
Hypertension	256 (43%)	246 (42%)	10 (63%)	0.104
Gout	29 (4.8%)	28 (4.8%)	1 (6.3%)	0.789
Recurrent UTIs	31 (5.2%)	27 (4.6%)	4 (25%)	<0.001
Bowel disease	133 (22%)	128 (22%)	5 (31%)	0.375
Chronic kidney disease	52 (8.7%)	48 (8.2%)	4 (25%)	0.019

On multivariable analysis (Table 3), patients were more likely to have a postoperative admission if they were older (OR 1.06; 95% CI 1.01–1.10, p = 0.01) or had a history of recurrent UTIs (OR 7.40, 95% CI 1.78–30.67, p = 0.006). No other factors correlated with postoperative admissions.

**TABLE 3:** Result of multivariable logistic regression model predicting readmission after URS.

	OR	95% CI	P
Age	1.06	1.01–1.10	0.01
Body mass index	0.95	0.87–1.03	0.222
Preoperative stone size	0.94	0.81–1.08	0.372
Gender	0.66	0.22–1.98	0.456
Preoperative ED visit	0.28	0.07–1.12	0.07
Pre Stent (yes)	2.01	0.55–7.39	0.289
Diabetes mellitus	0.49	0.11–2.22	0.357
Recurrent UTIs	7.40	1.78–30.67	0.006
Chronic kidney disease	1.42	0.33–6.19	0.639

## 4 DISCUSSION

This study provides further modern evidence of risk factors for readmission within 30 days of URS. Although some studies have described ED visits or readmission after urological procedures,^12^, ^14^ little evidence is available with regard to the prognostic factor of readmission after URS.^9^ Unplanned readmission is costly. Stephen et al estimated the cost to Medicare of unplanned re‐hospitalization in 2004 at $17.4 billion.^17^ Data to substantiate the magnitude and aetiology for post‐surgical complications, mortality, ED visits and readmission are therefore needed.

URS is a well‐established procedure for the treatment of urinary stone disease. Several studies have reported complication and mortality rates.^6^, ^7^, ^8^ The EAU guidelines estimated an overall post‐URS complication rate of 9–25%.^4^ Ureteral stent discomfort, ureteral wall injury and stone migration are the most common major complications; however, patients are also at risk for severe urosepsis and multi‐organ failure which can lead to death.^6^ To avoid severe complications, prompt recognition and treatment of these complications are necessary. By identifying the risk factors that increase the chance a patient will be readmitted after URS, we can better manage those patients at the highest risk for complications.

Bhojani et al showed in a systematic review and meta‐analysis that older age, comorbidities such as ischemic heart disease and diabetes mellitus, preoperative stent placement, positive urine culture and longer procedure time were associated with increased postoperative urosepsis risk.^8^ Bhanot et al reported that over 60% of all reported URS‐related deaths occurred in the elderly (>65 yr old).^6^ More deaths were in females than in males, despite urinary stone disease being more common in males. The authors noted that the female gender was a risk factor for developing adverse events after URS owing to the predisposition of women to UTIs. Our results also demonstrated that female patients were more likely to have recurrent UTIs than male patients, however, we showed no gender difference for UTIs after surgery in univariate analysis. On the other hand, age (68 vs 56 years of age) and history of recurrent UTIs were associated with readmission (gender was not an independent factor in readmission in multivariable analysis). These results indicate that patients with recurrent UTIs, regardless of gender, are more likely to need to be readmitted after URS. This finding aids in predicting complications and could help urologists provide tailored treatment recommendations for each urinary stone patient. Older patients with recurrent UTIs can choose extracorporeal shock wave lithotripsy or conservative therapy rather than URS if these are options. In addition, 43 % of patients who needed readmission had postoperative UTIs. In the case of higher‐risk patients, it may be prudent to tailor preoperative antibiotics based on preoperative urine culture and employ intraoperative stone cultures or renal pelvic urine cultures to determine bacterial pathogens in infectious complications after ureteroscopy. Whitehurst et al suggested prevention strategies included the use of prophylactic antibiotics and aggressive treatment of UTIs to avoid mortality from kidney stone disease.^18^ We should consider a longer period of preoperative and postoperative antibiotics if patients have a history of recurrent UTIs.

In our study, BMI, preoperative stent placement and DM were not associated with ED visits and readmission. Kelly et al described that some studies showed there was no significant difference between complication rates for normal‐weight patients and obese patients in their review.^19^ Furthermore, Bhanot et al showed five mortalities in obese patients (BMI > 25) of 72 patients' deaths in 15 articles.^6^ The mean BMI was higher (31 ± 7.9), which could be potentially introducing selection bias but, BMI was not significantly different between non‐admitted patients and admitted patients in our study. Preoperative stent placement was not associated with readmission and ED visits in our study, which is similar to the report by Mittakanti et al.^10^

Some studies reported that DM was associated with increased postoperative urosepsis risk.^8^, ^20^ In the present study, we detected only seven readmitted patients with UTIs, despite 23% of patients having DM, and DM was not an independent factor for readmission after URS. Some reviews show that prolonged operative time is associated with increased adverse events following URS.^6^, ^21^ However, our study showed that surgery time was not significantly different between those admitted vs. those not admitted after surgery.

Recent studies showed that urinary stone disease is likely to increase the risk of CKD.^22^, ^23^, ^24^ In contrast, no studies demonstrated CKD causes urinary stone disease or increases the risk of complications. In our study, patients admitted had a higher risk of having CKD than those not admitted, but there was no significant difference in multivariable analysis. This result might indicate that the cause of CKD is not relative to urinary stone disease, but DM, hypertension and other kidney diseases including chronic UTIs.

Witherspoon et al described 16% of patients receiving urological surgeries had an ED encounter and advocated for fewer unnecessary ED encounters to reduce the burden on strained systems.^25^ When evaluating 40 (7.1%) patients who visited ED, we could not detect factors to predict an ED visit. Interestingly, we identified the mean age in admitted patients is significantly higher than in not admitted patients who visited ED, and whether the patient visited ED preoperatively or not is a factor in visiting the ED postoperatively. This result indicates other multiple factors, including the patient's mental status, how easy it is to access ED and financial situation, related to the factor of ED visiting. Therefore, a different approach is required to assess and reduce ED visits.

There are several limitations to our study. First, we do not use a UAS. Traxer et al. showed that using UAS reduced the incidence of postoperative infectious complications.^26^ The use of ureteral access sheaths was expected to improve vision by establishing a continuous outflow and decrease intrarenal pressure; thus, it could affect the rate of readmission or ED visits. Second, the retrospective design of our study has limited statistical analyses. The cohort consisted of a selected population in order to have the same number in each gender, potentially posing a selection bias. Third, we could not investigate ED visits and readmissions outside of our institution. We might have underestimated the number of patients who visited the ED. Finally, although the symptoms of admitted patients were carefully evaluated, each subgroup was so small that we could not analyse the relationship between each symptom and factors of readmission. Large prospective studies could further clarify the predictors of readmission after URS.

## 5 CONCLUSION

This study demonstrated that advanced age and history of recurrent UTIs increased patients' risk of readmission following ureteroscopy. Older patients (>65) should be targeted for preoperative interventions to prevent readmission. These findings could help the risk of readmission and decide the kind of procedures. Further larger studies seem to result in reducing readmission to prevent complications of URS and cut the cost of health services.

## AUTHOR CONTRIBUTIONS

S.H. and M.K. wrote the original draft preparation. S.L., A.A. and E.S. performed the research. S.L. analysed the data. M.K. designed the research. S.N. supervised the research.

## DATA SHARING STATEMENT

The data sets generated and analysed during the current study are available from the corresponding author on reasonable request.

## DISCLOSURE OF INTEREST

All authors have nothing to disclose.

## Supporting information


**Table S1.** Comparison of the patients who did not visit the ED with patients who visited the ED.
**Table S2** **.** Comparison of the patients who visited ED and did not need admission with patients who needed admission.
