# Supplementary material for: Risk factors for readmission after ureteroscopy for stone disease: Modern single centre experience
Source: BJUI Compass. 2025 Mar 23;6(3):e70007. doi: 10.1002/bco2.70007 (PMC11930544; doi:10.1002/bco2.70007)
Supplement: Supplementary file 1 — Table S1. Comparison of the patients who did not visit the ED with patients who visited the ED. Table S2 . Comparison of the patients who visited ED and did not need admission with patients who needed admission. [file BCO2-6-e70007-s001.docx]

**Table S1. Comparison of the patients who did not visit the ED with patients who visited the ED.**

|  | **Overall**  **(n=600)** | **No ED visit within 30 days**  **(n=560)** | **Visited ED within 30 days**  **(n=40)** | **P** |
| --- | --- | --- | --- | --- |
| Age (years) | 56±15 | 56±15 | 56±19 | 0.877 |
| BMI (kg/m^2^) | 31±7.9 | 31±8 | 31±7.3 | 0.831 |
| Race  White  African American  Asian  Native American | 562 (94%)  15 (2.5%)  13 (2.2%)  6 (1.0%) | 523 (93%)  14 (2.5%)  13 (2.3%)  6 (1.1%) | 39 (98%)  1 (2.5%)  0 (0%)  0 (0%) | 0.702 |
| Preoperative stone size (mm) | 8.5±4.7 | 8.6±4.7 | 8.1±4 | 0.574 |
| Preoperative ED visit | 224 (37 %) | 206 (37%) | 18 (45%) | 0.299 |
| Pre stented  Pre-stented  Nephrostomy tube placed | 115 (19%)  11 (1.8%) | 108 (19%)  10 (1.8%) | 7 (18%)  1 (2.5%) | 0.918 |
| Comorbidities  Diabetes mellitus  Hypertension  Gout  Recurrent UTIs  Bowel disease  Chronic kidney disease | 134 (22%)  256 (43%)  29 (4.8%)  31 (5.2%)  133 (22%)  52 (8.7%) | 124 (22%)  241 (43%)  28 (5.0%)  27 (4.8%)  123 (22%)  46 (8.2%) | 10 (25%)  15 (38%)  1 (2.5%)  4 (10%)  10 (25%)  6 (15%) | 0.675  0.494  0.476  0.153  0.655  0.141 |

**Table S2. Comparison of the patients who visited ED and did not need admission with patients who needed admission.**

|  | **Overall**  **(n=40)** | **Visited ED**  **and not admitted (n=24)** | **Visited ED**  **and admitted**  **(n=16)** | **P** |
| --- | --- | --- | --- | --- |
| Age (years) | 56±19 | 48±17 | 68±15 | <0.001 |
| BMI (kg/m^2^) | 31±7.3 | 33±7.1 | 28±6.9 | 0.043 |
| Gender |  |  |  | 0.519 |
| Male | 20 (50%) | 11 (46%) | 9 (56%) |  |
| Female | 20 (50%) | 13 (54%) | 7 (44%) |  |
| Race |  |  |  | 0.215 |
| White | 39 (98%) | 24 (100%) | 15 (94%) |  |
| African American | 1 (2.5%) | 0 (0%) | 1 (6.3%) |  |
| Asian | 0 (0%) | 0 (0%) | 0 (0%) |  |
| Native American | 0 (0%) | 0(0 %) | 0 (0%) |  |
| Preoperative stone size (mm) | 8.1±4 | 8.4±4.6 | 7.8±3.1 | 0.688 |
| Preoperative ED visit | 18 (45%) | 15 (63%) | 3 (19%) | 0.006 |
| Pre stented |  |  |  | 0.067 |
| Pre-stented | 7 (18%) | 2 (8.3%) | 5 (31%) |  |
| Nephrostomy tube placed | 1 (2.5%) | 0 (0%) | 1 (6.3%) |  |
| Surgery time (minutes) | 37±16 | 34±16 | 42±16 | 0.114 |
| Comorbidities |  |  |  |  |
| Diabetes mellitus | 10 (25%) | 7 (29%) | 3 (19%) | 0.456 |
| Hypertension | 15 (38%) | 5 (21%) | 10 (63%) | 0.008 |
| Gout | 1 (2.5%) | 0 (0%) | 1 (6.3%) | 0.215 |
| Recurrent UTIs | 4 (10%) | 0 (0%) | 4 (25%) | 0.010 |
| Bowel disease | 10 (25%) | 5 (21%) | 5 (31%) | 0.456 |
| Chronic kidney disease | 6 (15%) | 2 (8.3%) | 4 (25%) | 0.148 |
